# Two New Red/Near-Infrared Ir(Ⅲ) Complexes with Reversible and Force-Induced Enhanced Mechanoluminescence

**DOI:** 10.3390/ma16134702

**Published:** 2023-06-29

**Authors:** Yuzhen Yang, Qin Zeng, Weiqiao Zhou, Junjie Jiang, Zihao Zhang, Song Guo, Yuanli Liu

**Affiliations:** Guangxi Key Laboratory of Optical and Electronic Materials and Devices, College of Materials Science and Engineering, Guilin University of Technology, Guilin 541004, China; yangyuzhen812@163.com (Y.Y.); zqinnn@163.com (Q.Z.); wangyi123zhou@163.com (W.Z.); jiejiangjjj0513@163.com (J.J.); 1020190104@glut.edu.cn (Z.Z.)

**Keywords:** ionic iridium(III) complexes, red/near-infrared phosphorescence, mechanoluminescence, force-induced enhanced emission

## Abstract

Two novel ionic red/near-infrared Ir(III) complexes (Ir1 and Ir2) were reasonably designed and prepared using 2-(1-isoquinolinyl)-9,10-anthraquinone as the main ligand and 4,4′-dimethyl-2,2′-bipyridyl and 4,4′-dimethoxy-2,2′-bipyridyl as the auxiliary ligands, respectively. Both complexes showed bright phosphorescence in solution (peak at 618 nm with a shoulder at 670 nm). Interestingly, the phosphorescence peak of two Ir(III) complexes showed a blue-shift of about 36 nm after being ground. Simultaneously, both complexes exhibited mechanical force-induced enhanced emission, and the intensity of the luminescence for Ir1 and Ir2 increased by around two times compared to the one before being ground, respectively. Powder X-ray diffraction (PXRD) and time-dependent density functional theory (TD-DFT) calculation were utilized to understand well the mechanism of this phenomenon and suggested that the destruction of the well-ordered crystalline nature and the decline in triplet-triplet annihilation maybe responsible for the pressure-induced blue-shift and the enhancement of the phosphorescence.

## 1. Introduction

In recent years, tremendous attention has been given to smart luminescent materials whose photophysical properties can be regulated by various external stimulus (temperature [1,2], light [3,4], force [5], electric field [6,7], magnetic field [8], pH [9], specific ions [10,11], etc.). Upon external stimulus, such as pressure, grinding, and stretching, organic luminogens may exhibit evident changes in emission color and intensity except for chemical reactions, which are called mechanochromic luminescent (MCL) materials [12,13]. These materials could be applied in pressure sensors, optical data recording, and storage devices [14,15,16]. 

Mechanochromic luminogens, as captivating materials, have been extensively studied based on numerous pure organic small molecules [17,18], some metal complexes [19,20], liquid crystalline materials [21], and polymers [21]. Tong et al. [22] developed two solution-processable small molecules based on triazatruxene, and the two complexes showed bright blue–green (483 nm) and green (498 nm) emissions, respectively. The emission color of both complexes changed to yellow after being ground in a mortar as the crystalline state changed to amorphous powder. Wang et al. [23] designed and synthesized two asymmetric, traditional β-diketones named TPED2F and TPED2T. TPED2T exhibited a more obvious wavelength shift compared to TPED2F after it was ground and whose maximum emission wavelength showed a red-shift of about 54 nm from 493 nm to 547 nm along with marked color change from cyan to yellow when irradiated with ultraviolet light. Furthermore, TPED2F exhibited reversible mechanochromic properties when being heated, and TPED2T showed irreversible properties when being heated or fumigated. However, most of the previously reported materials are fluorescent luminogens, and few phosphorescent mechanochromic luminogens were reported. The mechanism of mechanochromic phosphorescent behavior is still unclear, which limits the further exploitation of these materials. Phosphorescent transition-metal complexes, especially Ir(III) complexes, exhibit outstanding photophysical properties, such as abundant triplet excited-state, high luminescence quantum efficiency, and tunable phosphorescence wavelength [24,25,26,27], which have been successfully used in the construction of organic light-emitting diodes [28], biosensors [29,30], information encryption [31], optical data recording [32], and storage [33]. Recently, several mechanochromic phosphorescent Ir(III) complexes have been reported. Su [34] and coworkers presented a reversible tricolor (blue, green, and yellow) Ir(III) complex-based luminescent switch, which can be attributed to the transformation from a crystalline state to an amorphous state. In 2021, Zhu [35] and coworkers synthesized two Ir(III) complex isomers, which showed distinctly opposite mechanochromic luminescence despite the same physical phase transformation, and the emitting excited state dominated by triplet charge transfer (^3^CT) can be responsible for their mechanochromic behaviors. However, the mechanochromic behaviors of most reported Ir(III) complexes are in the range of blue to green or orange, the mechanochromic phosphorescent Ir(III) complexes with red to deep-red emission are extremely rare.

It is well-known that the photophysical properties of the complexes largely rely on the pattern of molecular stacking and the intermolecular interaction in the solid state, and thus, the switch of solid-state luminescence can be achieved by altering molecular arrangement through external stimuli [36,37,38]. Herein, large conjugated groups, such as anthraquinone and isoquinoline, were introduced into two ionic Ir(III) complexes as main ligands, and 4,4′-dimethyl-2,2′-bipyridine and 4,4′-dimethoxy-2,2′-bipyridyl were selected as auxiliary ligands to prepare red to near-infrared luminogens, respectively. The two complexes showed bright red to near-infrared emission in solution and solid state. Interestingly, the emission spectra of both complexes showed a blue-shift of about 36 nm after being ground. Simultaneously, both complexes exhibited mechanical force-induced enhanced emission. Then, powder X-ray diffraction (PXRD) and time-dependent density functional theory (TD-DFT) calculation were utilized to understand well the mechanism of this phenomenon. The results indicated that the destruction of well-ordered crystalline nature and the decline in triplet-triplet annihilation maybe responsible for the pressure-induced blue-shift and the enhancement of the phosphorescence. We believe that our work will provide a new platform for the future design of highly efficient MCL Ir(III) complexes.

## 2. Materials and Methods

All reagents and solvents used in this paper were obtained through commercial channels and were used without further purification. 

The detailed instrument models are described in Supplementary Materials.

## 3. Results and Discussion

### 3.1. Syntheses and Characterization

**Figure 1:**
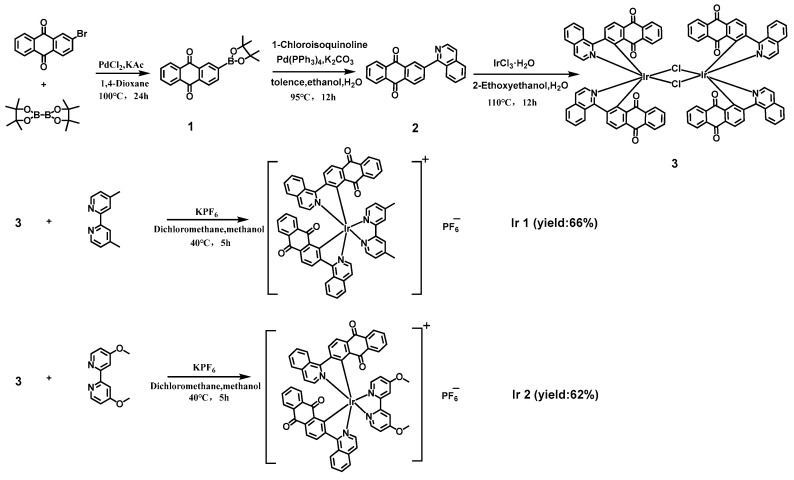
The synthesis of the two complexes.

Two compounds were designed and prepared as shown in Figure 1. First, the main ligand, 2-(1-isoquinolinyl)-9,10-anthraquinone, was synthesized using Suzuki coupling reaction with tetrakis(triphenylphosphine)palladium(0) as a catalyst. Next, dichloro-bridged diiridium complexes and complexes Ir1 and Ir2 were obtained according to the reported methods [39]. The chemical structures of all intermediates and products were confirmed using ^1^H nuclear magnetic resonance (NMR, 500 MHz), ^13^C NMR (126 MHz), ^31^P NMR (201 MHz), ^19^F NMR (472 MHz) spectra, and matrix-assisted laser desorption ionization time-of-flight mass spectrometry (MALDI-TOF MS). Furthermore, the photophysical properties of the two compounds were investigated using UV–Vis absorption spectrometry, and steady-state and transient phosphorescence spectrometry. From ^31^P NMR and ^19^F NMR, it was evident that the coupling constant between the nucleus of ^31^P and ^19^F can be calculated according to the heptet or doublet peak illustrated in the Supplementary Materials. Powder X-ray diffraction (XRD) was utilized to characterize the crystallinity of the two complexes. 

### 3.2. Photophysical Properties

**Figure 2:**
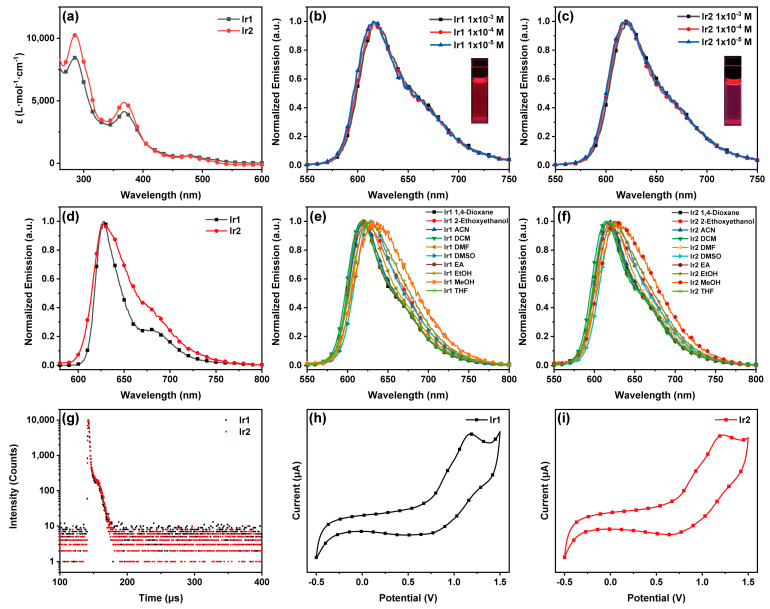
(**a**) Absorption spectra of Ir1 and Ir2 in CH_2_Cl_2_, (**b**,**c**) phosphorescence spectra of Ir1 and Ir2 at different concentrations in CH_2_Cl_2_, (**d**) low-temperature phosphorescence spectra (77 K) of Ir1 and Ir2 in CH_2_Cl_2_, (**e**,**f**) phosphorescence spectra of Ir1 and Ir2 in various solutions (1.0 × 10^−5^ M), (**g**) the decay curves of the luminescent lifetimes of the two complexes in degassed CH_2_Cl_2_, (**h**,**i**) cyclic voltammograms of Ir1 and Ir2 under a scan rate of 100 mV/s in CH_2_Cl_2_.

First, the photophysical properties of both complexes in solution were investigated. As depicted in Figure 2a, the UV–Vis absorption and emission spectra of Ir1 and Ir2 in dichloromethane (CH_2_Cl_2_) solution at room temperature were measured, and they exhibited almost the same peak pattern. The strong absorption peak below 315 nm may be attributed to the ligand-centered π-π* transition of 2-(1-isoquinolinyl)-9,10-anthraquinone, and the low-energy absorption band longer than 315 nm may be ascribed to the ^1^MLCT (metal-to-ligand charge transfer) and ^1^LLCT (ligand-to-ligand charge transfer) transitions [40].

Then, both complexes (Ir1 and Ir2) exhibited bright red emissions in CH_2_Cl_2_ solutions visible to the naked eye when excited at 450 nm and their phosphorescence spectra showed a similar profile. For both emissions, full width at half maximum of Ir1 and Ir2 was 60 nm. Next, the emission behaviors of the two complexes in degassed CH_2_Cl_2_ solution at different concentrations from 1.0 × 10^−3^ M to 1.0 × 10^−5^ M were investigated as shown in Figure 2b,c, and the maximum peaks were at about 618 nm. The results revealed that there was almost no or weak intermolecular interaction in dilute solutions. 

Moreover, the photoluminescence lifetimes of two complexes in CH_2_Cl_2_ solutions are listed in Table 1, and the lifetime of Ir2 is obviously shorter than Ir1. The lifetimes of two complexes, Ir1 and Ir2, with peaks at 618 nm were fitted to be 4.58 and 3.55 μs, respectively, and the decay curves (Figure 2g) exhibited single exponential function, demonstrating their triplet-emitting nature. Then, the phosphorescence spectra of the two complexes in various solvents (1,4-dioxane, 2-ethoxyethanol, acetonitrile, CH_2_Cl_2_, N,N-dimethyl formamide, dimethyl sulfoxide, ethyl acetate, ethanol, methanol, and tetrahydrofuran) with bubbled N_2_ are illustrated in Figure 2e,f. It can be clearly observed that the change in the maximum phosphorescence peak of Ir1 and Ir2 is associated with the change in the polarity of the solvent. For instance, the maximum phosphorescence peak for the two complexes in a solution of DMSO (628 nm) and methanol (638 nm) showed a red-shift of about 10–20 nm compared with that in CH_2_Cl_2_ (618 nm). The obvious shift suggested that the phosphorescent emission maybe derived from the mixture of LC excited-states and triplet metal-to-ligand charge transfer (^3^MLCT) [33].

The low-temperature luminescent spectra of the two complexes in CH_2_Cl_2_ solutions were measured at 77 K as shown in Figure 2d. The maximum emission peak of Ir1 and Ir2 is at 628 and 627 nm, respectively, showing a slight red-shift compared with that at room temperature, which may result from the increase in the degree of conjugation for the molecules at low temperature. Besides, Ir1 exhibited distinct fine structures of the vibronic band at the peak of 681 nm. The energy level of triplet excited state of Ir1 and Ir2 was calculated to be 1.97 and 1.98 eV, respectively, through the highest-energy vibronic bands from low-temperature luminescent spectra [35].

The electrochemical behaviors of the two complexes were studied using cyclic voltammetry (CV), and the redox potentials are listed in Table 1 and are shown in Figure 2h,i. Ir1 and Ir2 exhibited an irreversible redox process in CH_2_Cl_2_ solution with oxidation wave at 0.72 V and 0.69 V, respectively, which can be assigned to the oxidation of the Ir center [41,42]. The similar CV curves reveal that different ancillary ligands have a low effect on electrochemical behaviors.

**Figure 3:**
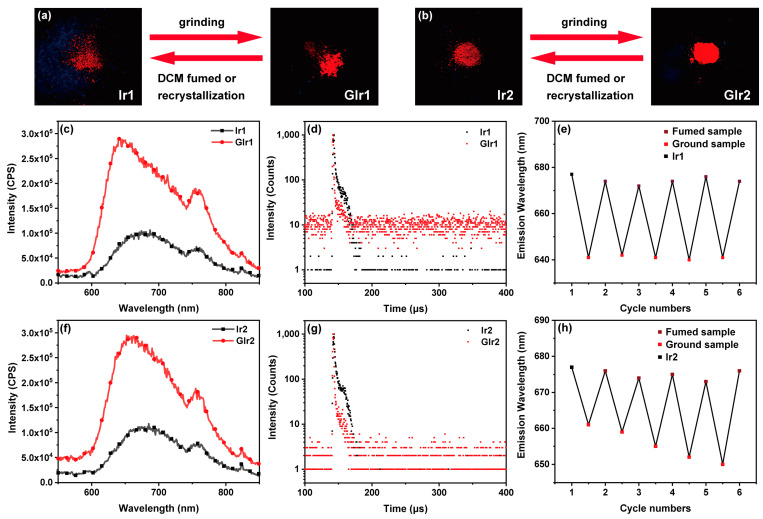
(**a**,**b**) Photographs of Ir1/GIr1 and Ir2/GIr2 upon irradiation with UV light. (**c**,**f**) Emission spectra of complexes Ir1, GIr1, Ir2, and GIr2 in solid states (the emission peak at ca. 750 nm may be caused by the laser). (**d**,**g**) Decay curves of complexes Ir1, GIr1, Ir2, and GIr2 in solid states. (**e**,**h**) Cycles of mechanochromism of Ir1 and Ir2. The letter “G” represents ground samples.

Next, the photophysical properties of Ir1 and Ir2 in solid state and their mechanochromic behaviors were investigated in detail. First, the emission spectra of the two complexes were measured (Figure 3c,f), and the numerical data are summarized in Table 1. The as-synthesized samples and ground samples were named Ir1 and GIr1, respectively (the same as Ir2 and GIr2). Both complexes exhibited deep-red emission visible to the naked eye when excited at 469 nm with the maximum phosphorescence peak at 677 nm. After being ground in a ceramic mortar, complex Ir1 showed a blue-shift from deep-red (λ_em_ = 677 nm) to red (λ_em_ = 641 nm), as depicted in Figure 3a,c. The similar mechanochromic behaviors can be clearly observed for complex Ir2, but Ir2 showed a relatively small force-induced spectral shift (16 nm) compared with Ir1 (Δλ_MCL_ = 36 nm) (Figure 3b,f). Impressively, two complexes exhibited pressure-induced luminescence enhancement, that is, they are MCL materials. The intensity of the luminescence for Ir1 and Ir2 increased by around two times compared to the one before being ground, respectively. Interestingly, the red luminescence can be easily reverted to near-infrared emission upon fuming with CH_2_Cl_2_, and the change of emission color in solid states can be repeated several times, suggesting distinguished reversibility in this process (Figure 3e,h). Furthermore, the photoluminescence decay times of Ir1 and Ir2 in solid state before and after being ground were studied, and the luminescent lifetimes of samples in different states were listed as shown in Table 1. It can be clearly seen that the luminescent lifetime (τ) of the as-synthesized sample and ground sample were definitely different, and both of them showed a time-resolved luminescent decay between the two different states. The blue-shift for the two complexes after being ground maybe due to the change of the intermolecular interaction or alteration of the pattern of solid-state molecular packing. π-π interaction between large conjugated groups, such as anthraquinone and isoquinoline, existed in Ir1 and Ir2 and may be weakened after being ground. Furthermore, the pressure-induced luminescence enhancement may result from the decline of the triplet-triplet annihilation before being ground.

### 3.3. Mechanochromic Luminescent Mechanism Investigations

**Figure 4:**
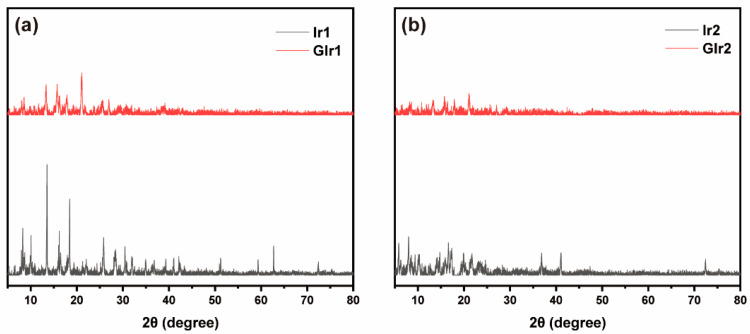
(**a**,**b**) PXRD patterns of as-synthesized sample (Ir1 and Ir2) and ground sample (GIr1 and GIr2).

In order to realize the possible origin of the MCL behaviors of these two compounds, the powder X-ray diffraction (PXRD) tests were carried out on the as-synthesized sample and ground sample. As illustrated in Figure 4, the intense and sharp reflection peaks can be observed in Ir1 and Ir2, suggesting that these two complexes are well-ordered and crystalline in nature. Moreover, the ground samples showed a weaker diffraction signal, suggesting that the original ordered structures were destroyed. Furthermore, the results showed that the crystallinity of Ir1 is higher than that of Ir2; thus, Ir1 showed an obvious blue-shift compared with Ir2 after being ground. In other words, MCL behaviors of the two iridium(III) complexes may be due to the phase transition from crystalline to amorphous states.

### 3.4. Theoretical Calculation

**Figure 5:**
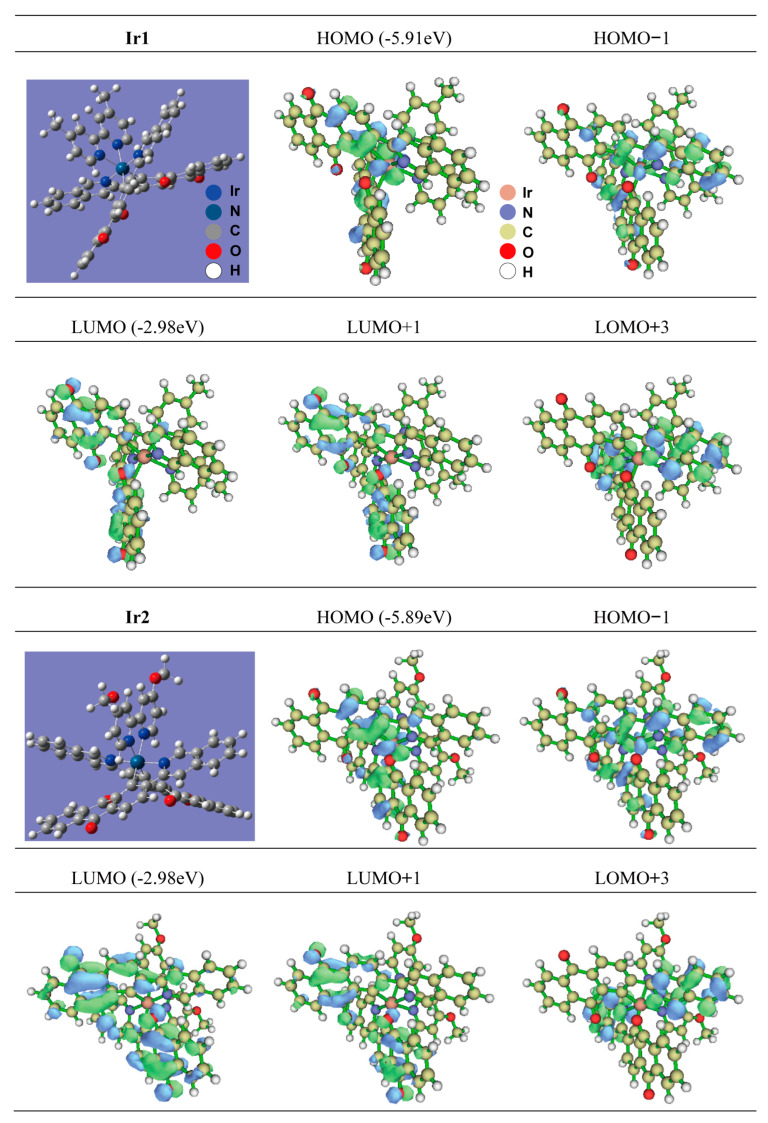
The optimized structures (blue background) and distributions of molecular orbitals (other graphs) of Ir1 and Ir2 (the calculation method is illustrated in the section of calculation method in the Supporting Information).

To understand well the underlying mechanism of photophysical properties of the two complexes, theoretical calculation was carried out using B3LYP and time-dependent density functional theory (TD-DFT) (Figure 5 and Table 2). The highest occupied molecular orbital (HOMO) and the lowest unoccupied molecular orbital (LUMO) energy levels of Ir1 and Ir2 are −5.97/−2.98 eV and −5.89/−2.98 eV, respectively. According to the optimized steric configuration, it can be seen that both Ir1 and Ir2 adopted twisted octahedral configuration. The frontier molecular orbitals (FMO) indicated a difference from the common iridium complex. The HOMO and HOMO-1 of the two complexes were located on the main ligand and iridium atom without the ancillary ligands, and the LUMO, LUMO+1, and LUMO+3 of the two complexes were located on the cyclometalating ligand. The triplet excited-states of the two complexes can be assigned to the mixture of MLCT transitions and LLCT transitions. Furthermore, the ancillary ligands were not involved in FMO construction, and this is probably a good explanation for the identical photophysical behaviors of Ir1 and Ir2 [43,44].

## 4. Conclusions

To sum up, two new cationic iridium(III) complexes with different auxiliary ligands were successfully designed and prepared. Both of them have reversible MCL behaviors, and the emission color can be tuned by grounding and fuming with a solution. Moreover, the MCL properties of Ir1 with methyl-bearing ancillary ligands are more remarkable. The transformation between crystalline and amorphous states and π-π interaction between large conjugated groups maybe the primary reasons for the MCL behavior. In addition, two complexes showed red photoluminescence in the solid and solution state. The different ancillary ligands play a functional role in their MCL behaviors but have a neglectable influence on the photophysical behaviors. We believe that our work will provide valuable guidelines for researchers to design and prepare novel phosphorescent MCL materials based on iridium(III) complexes in the future.

## Figures and Tables

**Table 1:** The photophysical data of Ir1 and Ir2.

Complexes	Emission in Degassed CH_2_Cl_2_		Emission in Solid States		E_g_ [eV] ^a^	E_onset_^ox^ [eV]	T_1_ [eV] ^b^
	λ_em_ [nm]	τ [μs]	λ_em_ [nm]	τ [μs]			
Ir1	616	4.58	677	2.21	2.01	0.72	1.97
GIr1	-	-	641	0.94	-	-	-
Ir2	620	3.55	677	1.86	2.00	0.69	1.97
GIr2	-	-	661	0.84	-	-	-

^a^ E_g_ can be calculated from absorption onset according to the UV–Vis spectra. ^b^ T_1_ = 1240/λ_77K_.

**Table 2:** The theoretical calculations of molecular orbital for the two complexes.

Complexes	State	HOMO	LUMO	Configuration	Character
		(eV)	(eV)		
Ir1	T_1_	−5.97	−2.98	HOMO−1→LUMO+1, 6.48%	LLCT
				HOMO−1→LUMO+3, 3.38%	MLCT/LLCT
				HOMO→LUMO, 84.5%	MLCT/LLCT
Ir2	T_1_	−5.89	−2.98	HOMO−1→LUMO+1, 5.78%	LLCT
				HOMO−1→LUMO+3, 2.88%	MLCT/LLCT
				HOMO→LUMO, 79.4%	MLCT/LLCT

## Data Availability

Raw data are available from the authors upon request.

## Supplementary Materials

The following supporting information can be downloaded at: https://www.mdpi.com/article/10.3390/ma16134702/s1, Figure S1: The ^1^H NMR spectrum of the main ligand 2-(1-isoquinolinyl)-9,10-anthraquinone. Figures S2–S6: The NMR spectra and MALDI-TOF spectrum of Ir1. Figures S7–S11: The NMR spectra and MALDI-TOF spectrum of Ir2. Figure S12. PXRD patterns of as-synthesized sample Ir1 and ground sample GIr1. Figure S13. PXRD patterns of as-synthesized sample Ir2 and ground sample GIr2. Reference [45] is cited in the Supplementary Materials.
