# Supplementary material for: Two New Red/Near-Infrared Ir(Ⅲ) Complexes with Reversible and Force-Induced Enhanced Mechanoluminescence
Source: Materials (Basel). 2023 Jun 29;16(13):4702. doi: 10.3390/ma16134702 (PMC10342365; doi:10.3390/ma16134702)
Supplement: Supplementary file 1 [file materials-16-04702-s001.zip › materials-2393158-supplementary.pdf]

materials

MDPI

Supporting Information

Two New Red/Near-Infrared Ir(III) Complexes with Reversible and Force-Induced Enhanced Mechanoluminescence

Yuzhen Yang, Qin Zeng, Weiqiao Zhou, Junjie Jiang, Zihao Zhang, Song Guo * and Yuanli Liu *

Guangxi Key Laboratory of Optical and Electronic Materials and Devices, College of Materials Science and Engineering, Guilin University of Technology, Guilin 541004, China; yangyuzhen812@163.com (Y.Y.); zqinnn@163.com (Q.Z.); wangyi123zhou@163.com (W.Z.); jiejiangjjj0513@163.com (J.J.); 1020190104@glut.edu.cn (Z.Z.)

* Correspondence: bobingjin@glut.edu.cn (S.G.); lyuanli@glut.edu.cn (Y.L.)

1. General Experimental Information

The NMR spectra were measured on Bruker Avance 500 MHz with tetramethylsilane as the internal standard. Mass spectra were obtained on Bruker matrix-assisted laser desorption/ionization time-of-flight mass pectrometer (MALDI-TOF-MASS). The UV–Vis absorption spectrum was recorded on a Lambda 750 spectrometer (PerkinElmer, America). Emission spectra and lifetimes were measured on a FluoroMax-4 fluorescence spectrophotometer (Horiba, Japan). The excited-state lifetime was measured on a transient spectro-fluorimeter (Edinburgh QM 8000) with a time-correlated single-photon counting technique. Photographs were taken using Canon 90D camera (Canon, Japan). Cyclic voltammetry was performed on the IVIUMSTAT.h instrument with a scan rate of 100 mV s^{-1} in CH_2Cl_2 solutions, a glassy-carbon electrode as the working electrode, an aqueous saturated potassium chloride electrode as the pseudo-reference electrode, and a platinum wire as the counter electrode, respectively. A 0.1 M solution of tetrabutylammonium hexafluorophosphate in CH_2Cl_2 was used as the supporting electrolyte, and the ferrocene/ferrocenium (Fc^+/Fc) potential was measured and selected as the internal standard. Powder X-ray diffraction of original and mechanically ground forms were measured in PANalytical diffractometer.

2. Calculation method

The ground-state geometrical configuration was optimized by density functional theory (DFT) with B3LYP functional. Based on the optimized ground state molecular structure, the time-dependent DFT (TDDFT) approach associated with the polarized continuum model (PCM) in dichloromethane media was carried out to

obtain the vertical excitation energies of triplet states (Tn). The calculation was performed using the Gaussian 16 B.01 suite of programs. The SDD basis set was used to treat the iridium atom, whereas the 6-31G* basis set was used to treat all other atoms. The contours of the highest occupied molecular orbital (HOMO) and lowest unoccupied molecular orbital (LUMO) were plotted using Multiwfn 3.8 soft. [S1-2]

3. Synthesis of cyclometalating ligand and Iridium(III) Complexes

Synthetic routes of **Ir1** and **Ir2** are described in **Figure 1**.

Synthesis of main ligand **2**:

2-Boronate anthraquinone (1). 2-bromoanthraquinone (2.0 g, 7.0 mmol), bis(pinacolato)diboron (2.1 g, 7.0 mmol), and [1,1'-bis(diphenylphosphino)ferrocene]dichloropalladium(II) (complex with dichloromethane) (0.3 g, 0.35 mmol) were dissolved in 30 mL 1,4-dioxane, and 10 equivalent KAc was added. The mixture was refluxed at 100°C under a nitrogen atmosphere for 24 h. After that, the resulting precipitate was discarded using filtration, and the filtrate was extracted with CH_2Cl_2 and water several times. Then, the organic layer was dried over anhydrous Na_2SO_4, filtered, and concentrated. Finally, the residue was purified using column chromatography (silica, dichloromethane/petroleum ether = 1:2) and yielded a pale-yellow powder **1** (yield: 70%). ^{1}H NMR (500 MHz, Chloroform-d) δ 8.75 (s, 1H, arom. C_6H_3), 8.36 – 8.26 (m, 3H, arom. C_6H_4), 8.20 (d, J = 7.6 Hz, 1H, arom. C_6H_3), 7.84 – 7.76 (m, 2H), 1.38 (s, 13H, CH_3 groups).

2-(1-isoquinolinyl)-9,10-anthraquinone (2). The precursor 2-boronate anthraquinone (1.0 g, 3.0 mmol), 1-chloroisoquinoline (0.5 g, 3.0 mmol), and tetrakis(triphenylphosphine)palladium (0.1 g, 0.09 mmol) were dissolved in the mixture of toluene, ethanol, and water (V:V:V = 3:1:1, 40 mL), and 10 equivalent K_2CO_3 was added. The mixture was refluxed at 95°C under a nitrogen atmosphere for 12 h. The post-processing was similar to that of **1.** The yellow product **2** was collected and dried overnight (yield: 70%). ^{1}H NMR (500 MHz, Chloroform-d) δ 8.68 (d, J = 8.6 Hz, 2H), 8.51 (d, J = 7.9 Hz, 1H), 8.42 – 8.29 (m, 2H), 8.19 (d, J = 8.0 Hz, 1H), 8.06 (d, J = 8.5 Hz, 1H), 7.96 (d, J = 8.1 Hz, 1H), 7.87 – 7.81 (m, 2H), 7.76 (t, J = 5.4 Hz, 2H), 7.61 (t, J = 7.6 Hz, 1H).

Synthesis of the chloro-bridged dimer **3**:

The dimer **3** was synthesized from a reaction of $IrCl_3 \cdot 3H_2O$ (0.26 g, 0.75 mmol) with 2-(1-isoquinolinyl)-9,10-anthraquinone (0.5 g, 1.5 mmol) in the mixture of 2-ethoxyethanol and water mixture (V:V = 3:1, 40 mL)

at 110°C under nitrogen atmosphere for 24 h. After cooling to room temperature, excess water was added into the mixture to induce precipitation. The precipitate was filtered and dried overnight.

Synthesis of Iridium(III) Complex:

The synthesis of **Ir1** and **Ir2** is according to a previously reported method. [S3-6]

*Synthesis and characterization of complex **Ir1***. Chloro-bridged dimer **3**(0.1 g, 0.08 mmol) and 4,4'-dimethyl-2,2'-bipyridyl (0.03 g, 0.16 mmol) were dissolved in the mixture of dichloromethane and methanol (V:V= 3:1, 32 mL), and 5 equivalent KPF_6 was added, and then the solution was refluxed at 40°C for 5 h. The mixture was extracted with CH_2Cl_2 and water several times to collect the organic phase that was dried over anhydrous Na_2SO_4, filtered, and concentrated. The crude product was purified with column chromatography (neutral alumina, dichloromethane/methanol = 100:1) to yield a deep-red powder **Ir1** (yield: 66%). ^{1}H NMR (500 MHz, Chloroform-d) δ 9.91 (s, 2H, arom. C_5H_2), 9.23 (s, 2H, arom. C_5H_2), 9.11 (d, J = 8.3 Hz, 2H), 8.28 (d, J = 7.6 Hz, 2H), 8.06 (s, 4H), 7.98 (t, J = 7.05 Hz, 2H), 7.93 (t, J = 7.05 Hz, 2H), 7.75 (t, J = 7.45 Hz, 2H), 7.70 (t, J = 7.55 Hz, 2H), 7.60 (m, J = 7.6 Hz, 4H), 7.55 (d, J = 6.2 Hz, 2H), 7.13 (d, J = 5.1 Hz, 2H), 2.71 (s, 6H. CH_3 groups). ^{13}C NMR (126 MHz, Chloroform-d) δ 183.26, 183.18, 166.30, 163.68, 155.48, 154.01, 151.79, 148.57, 140.27, 137.32, 134.19, 133.99, 133.76, 133.30, 132.67, 132.43, 130.35, 129.69, 129.14, 129.03, 128.17, 127.16, 127.03, 126.93, 126.30, 124.45, 29.70, 21.56. ^{19}F NMR (471 MHz, Chloroform-*d*) δ -72.83 (d, J = 711.21 Hz) ^{31}P NMR (202 MHz, Chloroform-*d*) δ-144.67 (sep, *J*= 713.06 Hz). MALDI-TOF-MS (m/z)： calcd. for $C_{58}H_{36}IrN_4O_4$, 1045.17； found, 1044.752. The related complex **Ir2** was prepared using similar procedures.

Ir2: deep-red powder (62% yield). ^{1}H NMR (500 MHz, Chloroform-d) δ 9.35 (s, 2H, arom. C_5H_2), 9.23 (s, 2H, arom. C_5H_2), 9.11 (d, J = 8.5 Hz, 2H), 8.27 (d, J = 7.5 Hz, 2H), 8.06 (t, J = 6.7 Hz, 4H), 7.98 (t, J = 8.2 Hz, 2H), 7.94 (t, J = 7.25 Hz, 2H), 7.74 (t, J = 7.4 Hz, 2H), 7.70 (t, J = 7.3 Hz, 2H), 7.63 (m, 4H), 7.50 (d, J = 6.4 Hz, 2H), 6.79 (d, J = 5.8 Hz, 2H), 4.41 (s, 6H, OCH_3 groups). ^{13}C NMR (126 MHz, Chloroform-d) δ 183.27, 183.16, 168.96, 166.34, 164.17, 158.10, 151.85, 149.55, 140.33, 137.27, 134.15, 133.95, 133.74, 133.27, 132.60, 132.37, 130.28, 129.70, 129.01, 128.16, 128.13, 127.12, 126.98, 126.89, 126.28, 124.37, 116.80, 112.13, 59.17, 30.94, 29.69, 29.61, 29.52, 29.31, 29.20, 21.22, 14.18. ^{19}F NMR (471 MHz, Chloro-form-*d*) δ -72.64 (d, J = 711.21 Hz). ^{31}P NMR (202 MHz, Chloroform-*d*) δ -144.44 (sep, *J*= 711.04 Hz) MALDI-TOF-MS (m/z): calcd. for $C_{58}H_{36}IrN_4O_6$, 1077.18； found, 1076.729.

References

[S1] Frisch, M.J.; Trucks, G.W.; Schlegel, H.B.; Scuseria, G.E.; Robb, M.A.; Cheeseman, J.R.; Scalmani, G.; Barone, V.; Petersson, G.A.; Nakatsuji, H.; Li, X.; Caricato, M.; Marenich, A.V.; Bloino, J.; Janesko, B.G.; Gomperts, R.; Mennucci, B.; Hratchian, H.P.; Ortiz, J.V.; Izmaylov, A.F.; Sonnenberg, J.L.; Williams, Ding, F.; Lipparini, F.; Egidi, F.; Goings, J.; Peng, B.; Petrone, A.; Henderson, T.; Ranasinghe, D.; Zakrzewski, V.G.; Gao, J.; Rega, N.; Zheng, G.; Liang, W.; Hada, M.; Ehara, M.; Toyota, K.; Fukuda, R.; Hasegawa, J.; Ishida, M.; Nakajima, T.; Honda, Y.; Kitao, O.; Nakai, H.; Vreven, Y.; Throssell, K.; Montgomery J, J.A.; Peralta, J.E.; Ogliaro, F.; Bearpark, M.J.; Heyd, J.J.; Brothers, E.M.; Kudin, K.N.; Staroverov, V.N.; Keith, T.A.; Kobayashi, R.; Normand, J.; Raghavachari, K.; Rendell, A.P.; Burant, J.C.; Iyengar, S.S.; Tomasi, J.; Cossi, M.; Millam, J.M.; Klene, M.; Adamo, C.; Cammi, R.; Ochterski, J.W.; Martin, R.L.; Morokuma, K.; Farkas, O.; Foresman, J.B.; Fox, D.J.; Wallingford. CT. 2016.

[S2] Lu, T.; Chen F. W. Multiwfn. J. Comput. Chem. 2012, 33, 580-592.

[S3] Guo, S.; Guo, C. X.; Lu, Z.; Du, L. L.; Gao, M.; Liu, S. J.; Liu, Y. L.; Zhao, Q. Crystals. 2021, 11, 1190.

[S4] Sun, H. B.; Liu, S. J.; Lin, W. P.; Zhang, K. Y.; Lv, W.; Huang, X.; Huo, F. W.; Yang, H. R.; Jenkins, G.; Zhao, Q.; Huang, W.. Nat. Commun. 2014, 5, 3601.

[S5] Tao, P.; Li, W. L.; Zhang, J.; Guo, S.; Zhao, Q.; Wang, H.; Wei, B.; Liu, S. J.; Zhou, X. H.; Yu, Q.; Xu, B. S.; Huang, W. Adv. Funct. Mater. 2016, 26, 881.

[S6] Nonoyama, M. Bull. Chem. Soc. Jpn. 1974, 47, 767.

NMR and MS Spectra

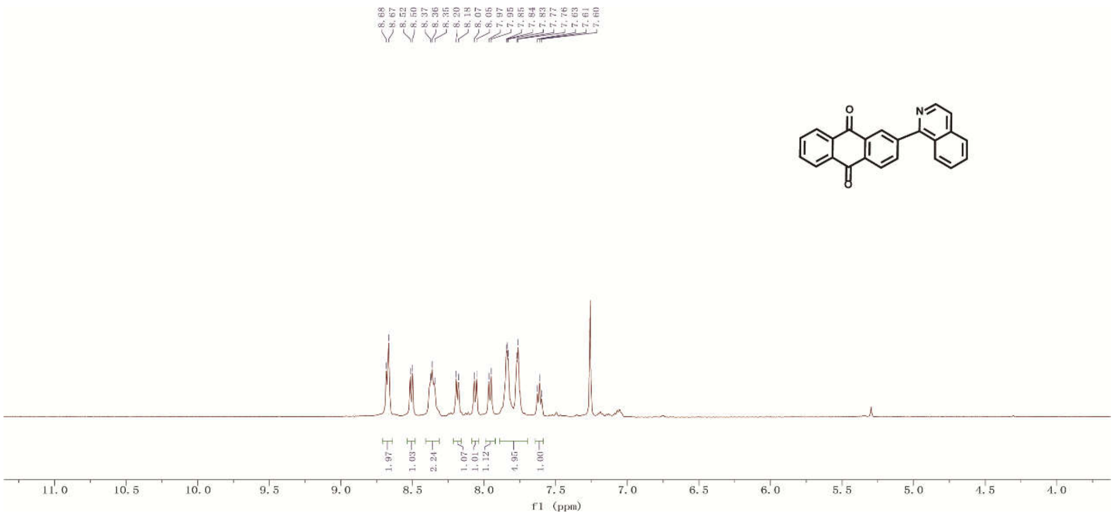

Figure S1. The ^{1}H NMR spectrum of the main ligand **2**.

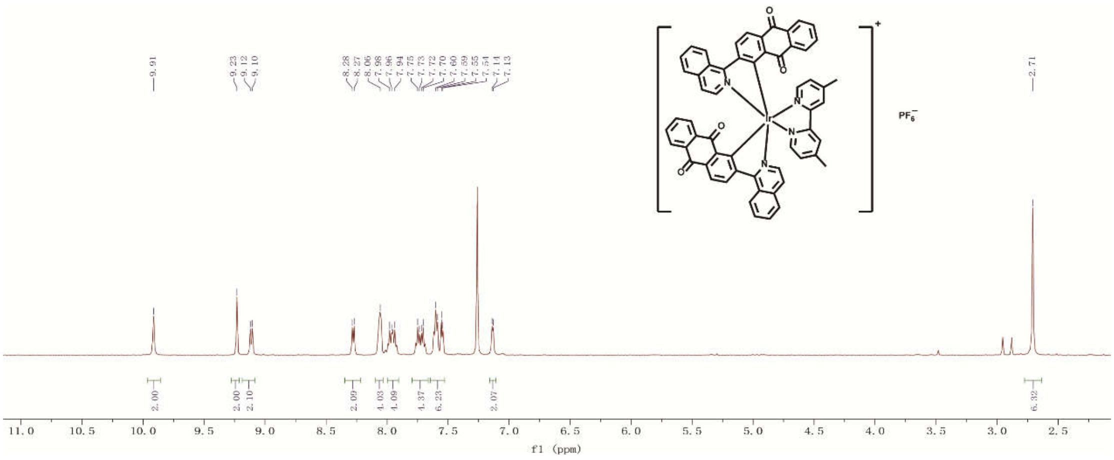

Figure S2. The ^{1}H NMR spectrum of complex **Ir1**.

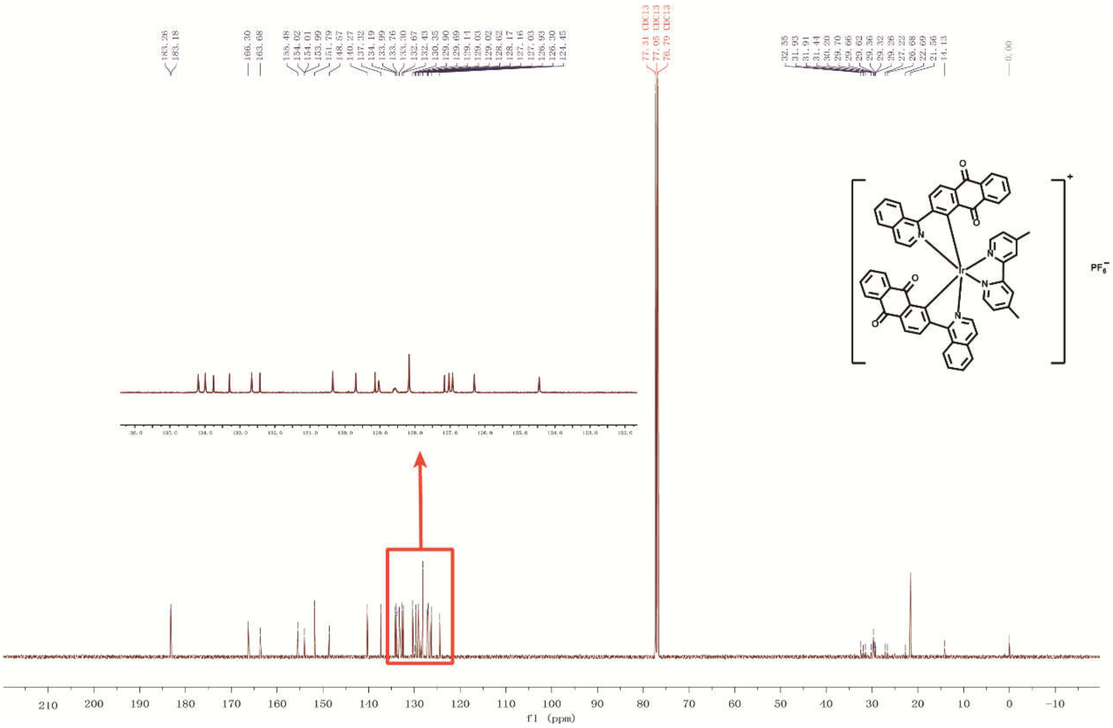

Figure S3. The ^{13}C NMR spectrum of complex **Ir1**.

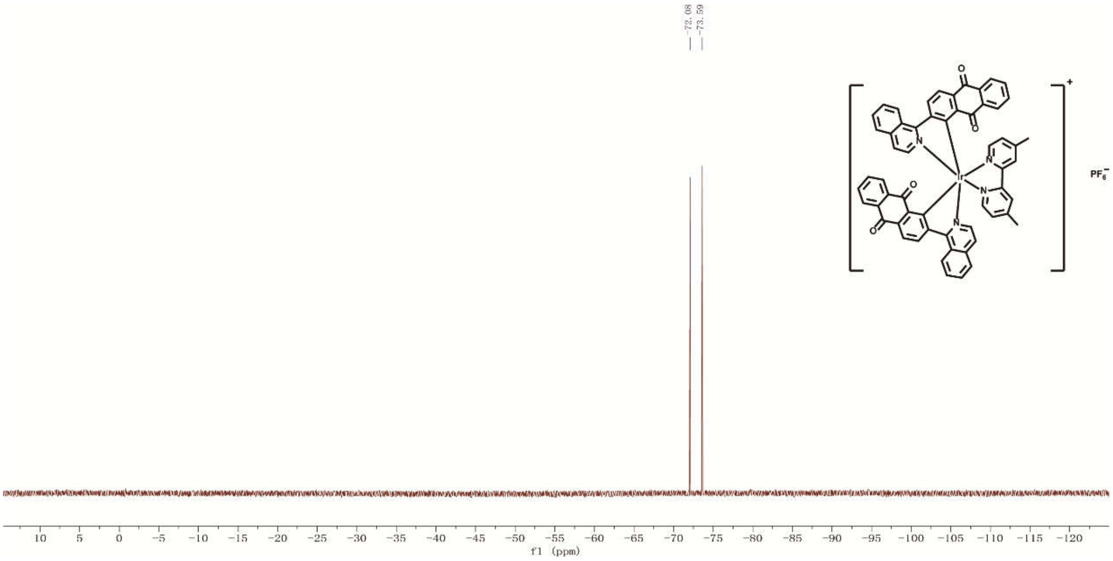

Figure S4. The ^{19}F NMR spectrum of complex **Ir1**.

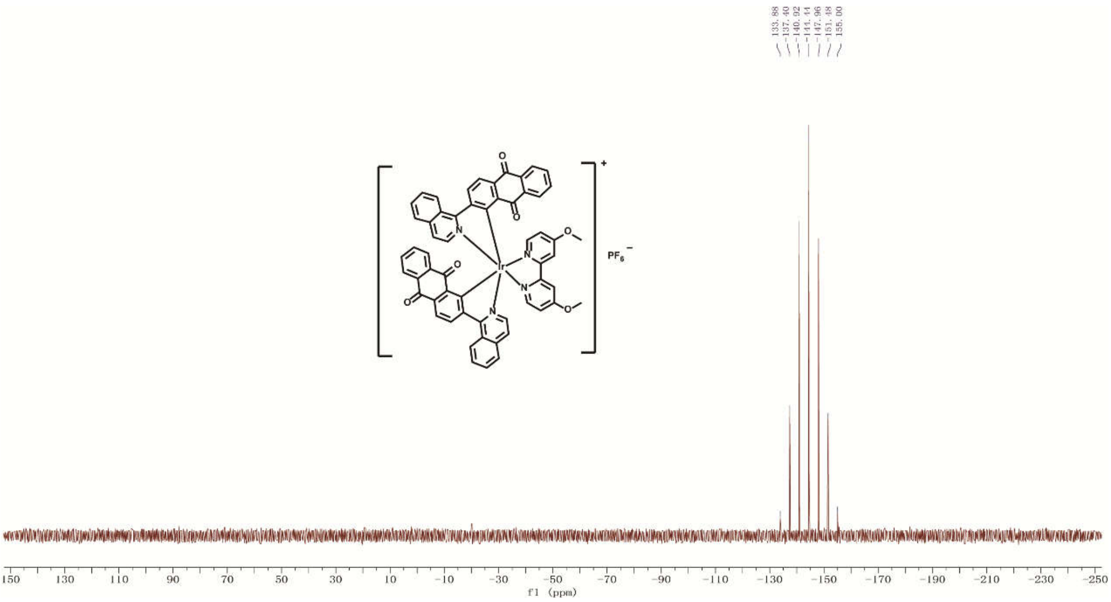

Figure S5. The ^{31}P NMR spectrum of complex **Ir1**.

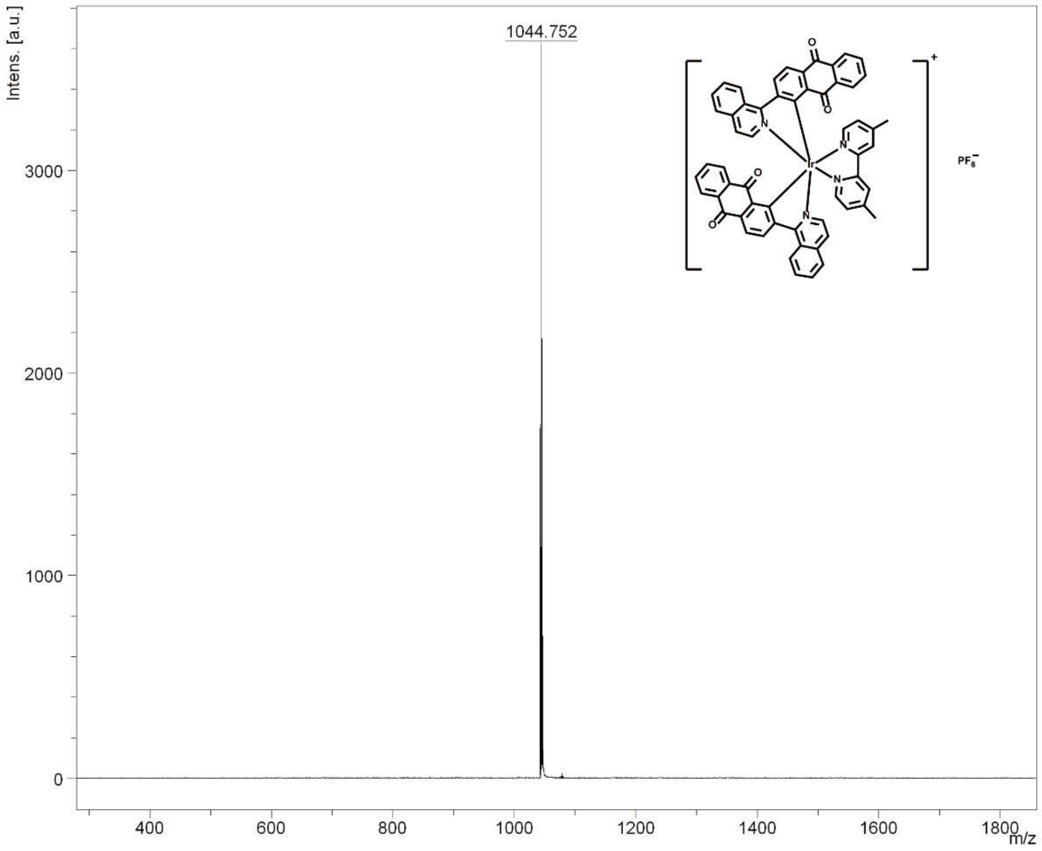

Figure S6. MALDI-TOF spectrum of complex **Ir1**.

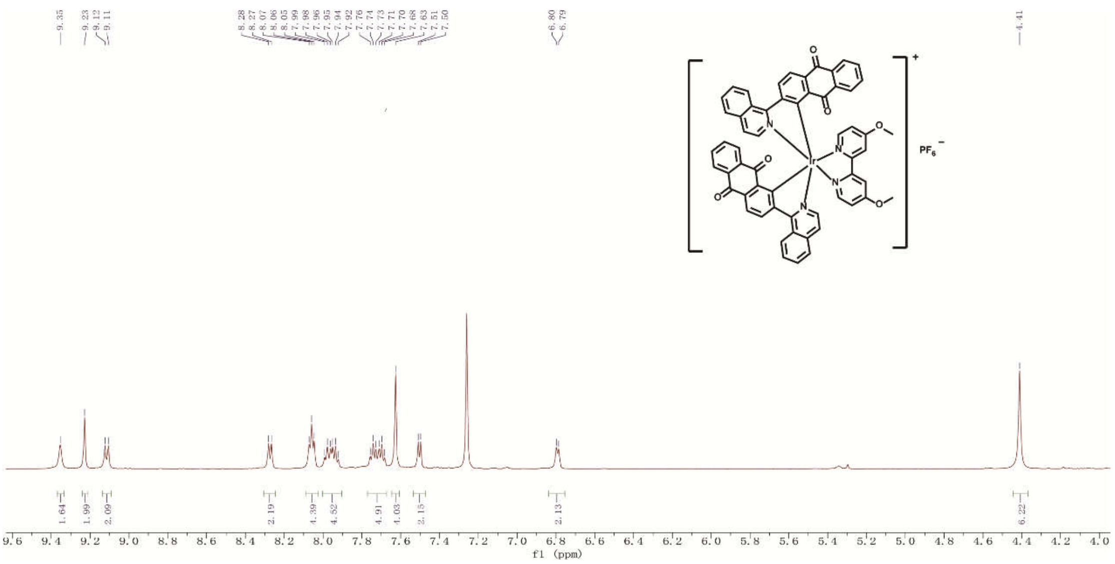

Figure S7. The ^{1}H NMR spectrum of complex **Ir2**.

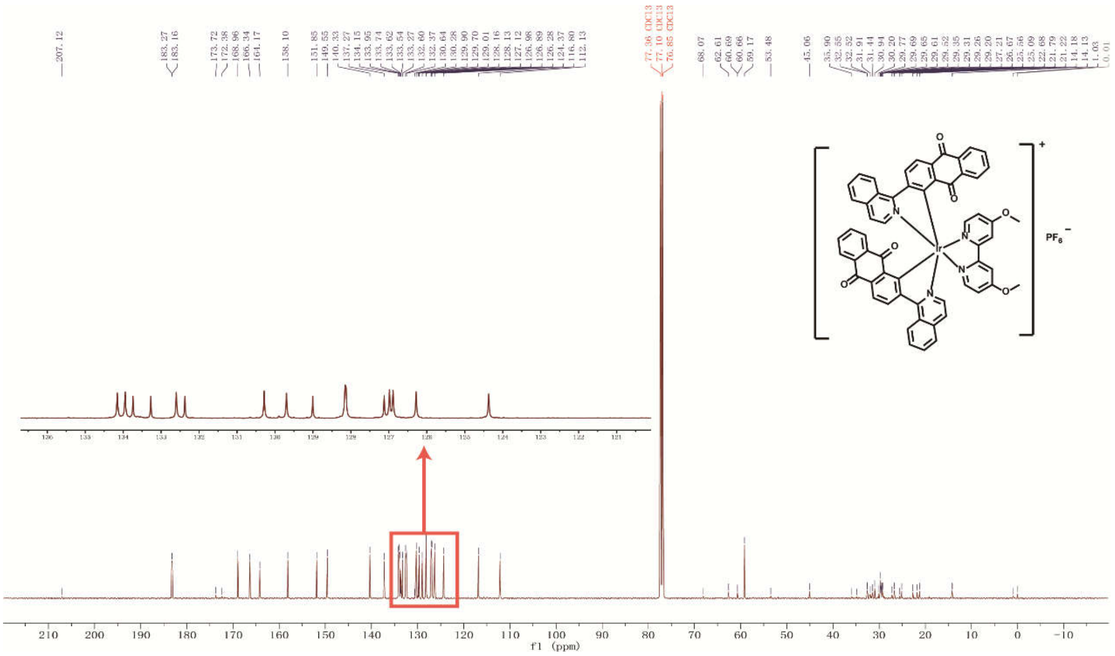

Figure S8. The ^{13}C NMR spectrum of complex **Ir2**.

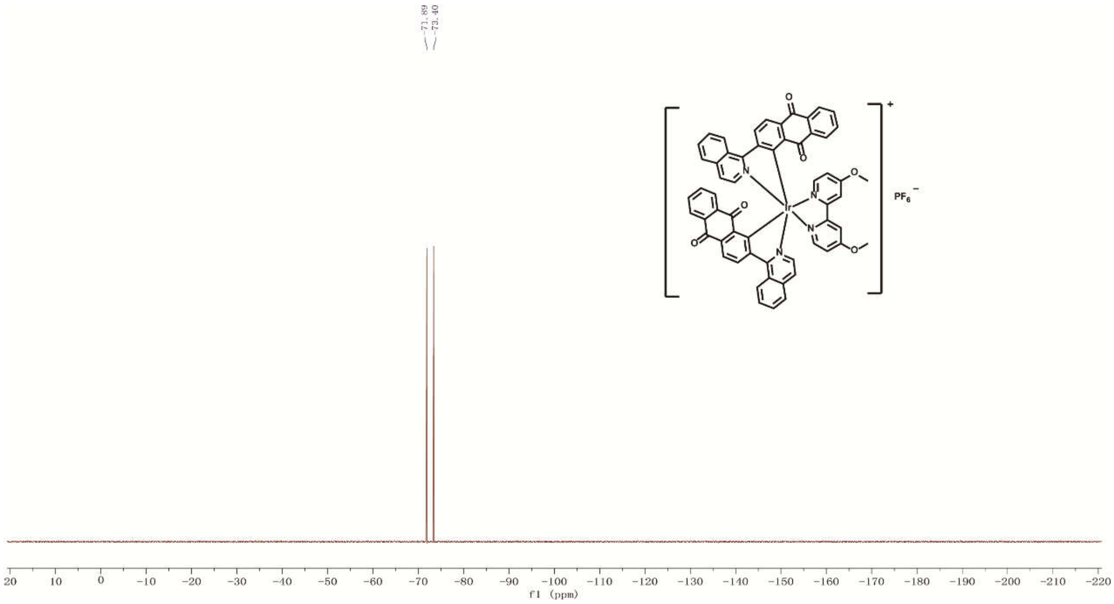

Figure S9. The ^{19}F NMR spectrum of complex **Ir2**.

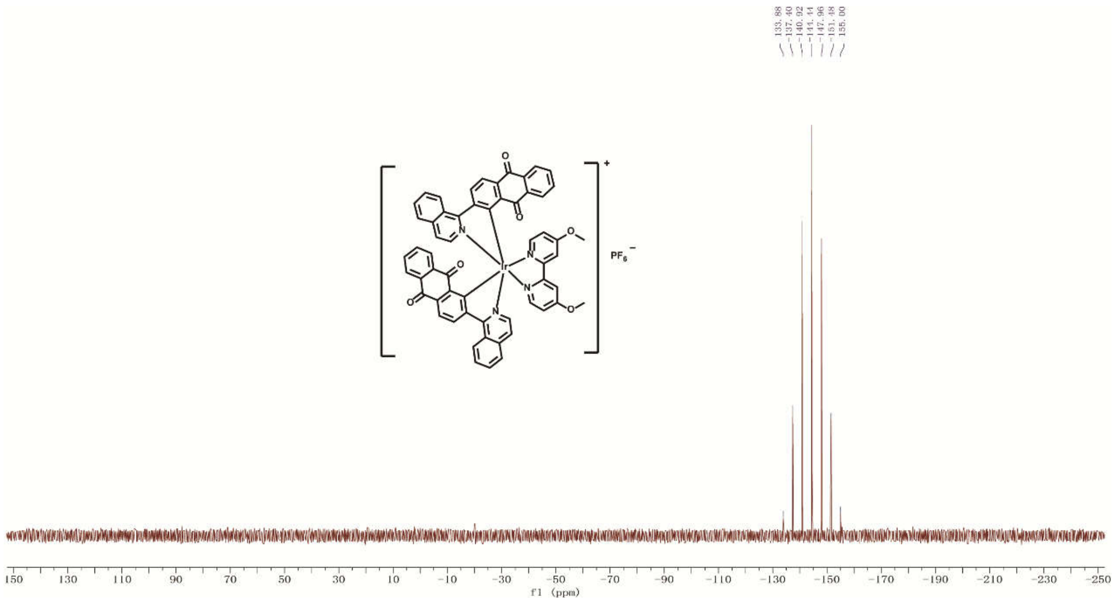

Figure S10. The ^{31}P NMR spectrum of complex **Ir2**.

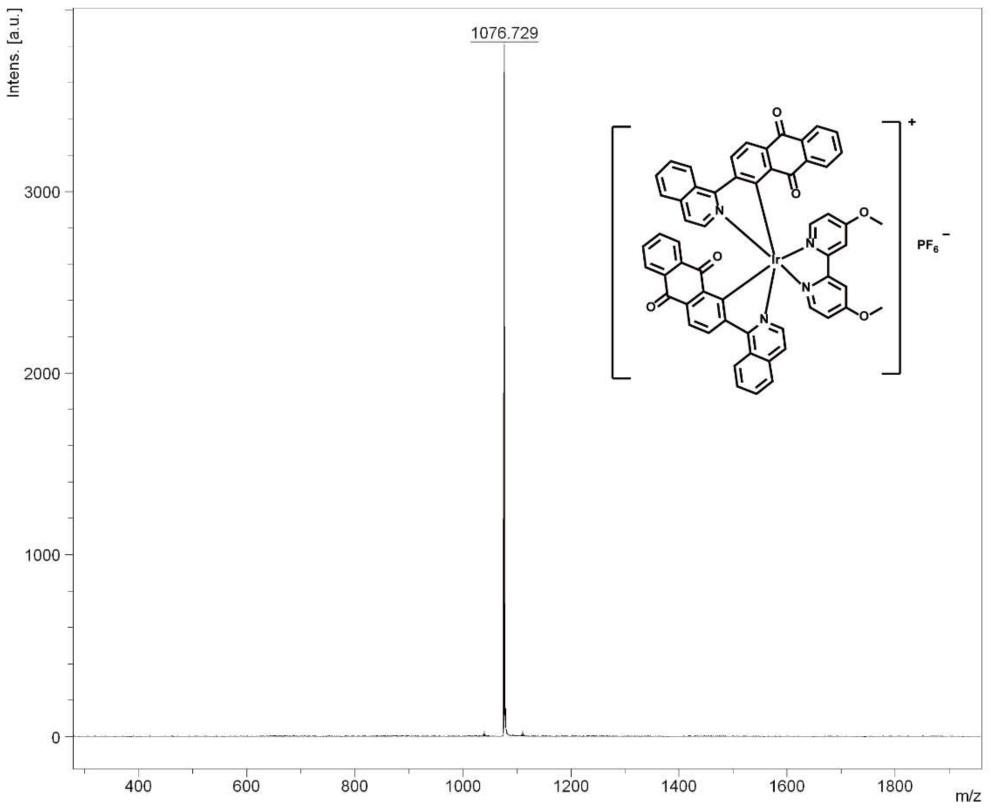

Figure S11. MALDI-TOF spectrum of complex **Ir2**.

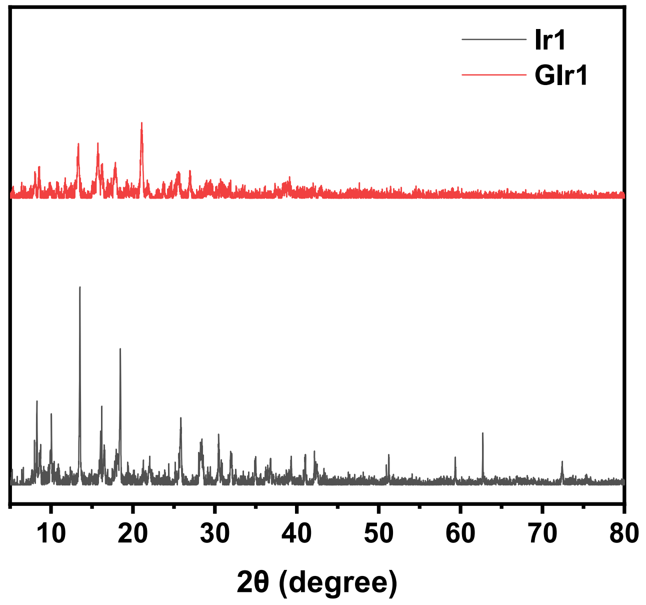

Figure S12. PXRD patterns of as-synthesized sample **Ir1** and ground sample **GIr1**.

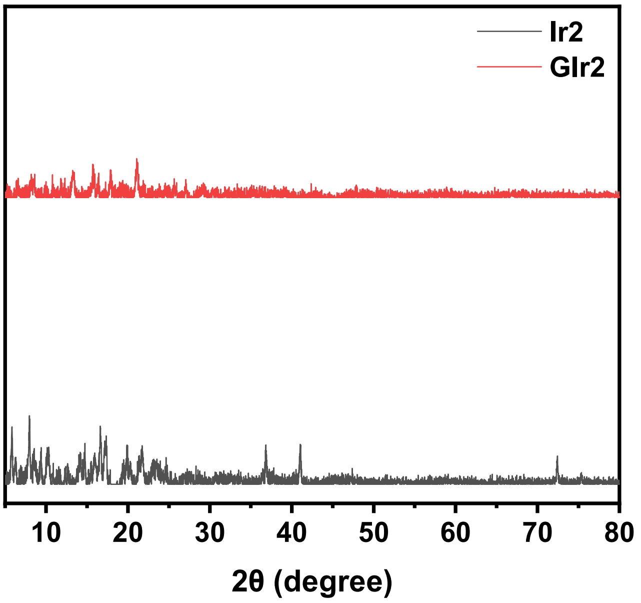

Figure S13. PXRD patterns of as-synthesized sample **Ir2** and ground sample **GIr2**.
